# Additive Manufacturing of Thermoset Elastomer–Thermoplastic Composites Using Dual-Extrusion Printing

**DOI:** 10.3390/polym17131800

**Published:** 2025-06-28

**Authors:** Nathalia Diaz Armas, Geet Bhandari, Stiven Kodra, Jinde Zhang, David Kazmer, Joey Mead

**Affiliations:** Department of Plastics Engineering, University of Massachusetts Lowell, Lowell, MA 01854, USA; geetbhandari2000@gmail.com (G.B.); stiven_kodra@student.uml.edu (S.K.); jinde_zhang@uml.edu (J.Z.); david_kazmer@uml.edu (D.K.); joey_mead@uml.edu (J.M.)

**Keywords:** multi-material additive manufacturing, fully compounded rubber, thermoplastics, 3D printing, adhesion strength

## Abstract

This work investigated the 3D printing of fully compounded thermoset elastomers using a custom-designed printer capable of processing both thermoplastics and elastomers containing fillers and specific cure packages. The adhesion strength between selected thermoset elastomers and thermoplastic combinations was studied, and the influence of key process parameters on adhesion was evaluated. The results showed that interfacial bonding was favored by the proximity of solubility parameters, the amorphous morphology of the thermoplastic, and increased chain mobility at the processing temperature. Rubber processing parameters significantly influenced adhesion, showing that curing at a lower temperature for a longer duration yielded better results than shorter, higher-temperature cures. Elemental analysis revealed the presence of rubber-specific components on the thermoplastic surface, suggesting interfacial migration. These findings contribute to advancing multi-material 3D printing by enabling the integration of rubber-like materials with thermoplastics, expanding opportunities for applications in high-temperature and chemically demanding environments.

## 1. Introduction

Additive manufacturing (AM) has become a fundamental tool for industry and academia to combine the potential of customized products and specialized material properties [1]. A broad range of materials are processed through AM techniques including metals, ceramics, and polymers [2]. In particular, elastomers are a unique class of polymer that have very high elongation coupled with good elastic recovery [3]. A variety of 3D printing techniques have been used to produce elastomer materials, including vat photopolymerization, inkjet printing, and direct ink writing [4]. However, many of these methods require using solvents, photocurable formulations, or specific rheological modifiers to enable the printing of elastomers. Among several major AM techniques, fused deposition modeling (FDM), where a thermoplastic polymer is supplied in filament form through a nozzle and then extruded, has become one of the most affordable and accessible processes [5,6]. While thermoplastic elastomers are often printed using FDM, this process presents significant limitations in printing flexible materials with low hardness, primarily due to filament buckling during extrusion [7].

While the printing of thermoplastic elastomers and some low-viscosity reactive elastomers has been successfully accomplished, very few articles on AM of fully compounded thermoset elastomers or traditional rubber compounds are reported in the literature. Fully compounded elastomers (typically comprised of the elastomer, filler, and cure package) are materials that are traditionally used for application-specific products. The challenges in printing these materials include the high viscosity, shrinkage, and thermosetting nature of the rubber. Despite this, the ability to print these materials is of industrial interest. Recently, the ability to print these materials has been investigated, and scientific reports have listed some AM processes to print nitrile butadiene rubber (NBR), ethylene propylene diene monomer (EPDM), and fluoroelastomer compounds [8,9,10,11,12].

Multi-material additive manufacturing (MMAM) expands the capabilities of traditional 3D printing by enabling the integration of dissimilar materials (such as rigid thermoplastics, flexible elastomers, and thermosets) into a single part without the need for assembly or adhesives [13,14]. This capability allows for the creation of complex, functional structures with tailored properties for applications in biomedical devices, soft robotics, wearables, and aerospace [15,16]. For example, Rahmatabadi et al. [17] demonstrated that dual-layer composite structures fabricated through MMAM can achieve shape memory behavior, illustrating how interfacial design can directly enable responsive functionalities. Common polymeric materials used in MMAM include PLA (polylactic acid), ABS (acrylonitrile butadiene styrene), PETG (polyethylene terephthalate glycol), and PEEK (polyether ether ketone), often paired with TPEs (thermoplastic elastomers) such as TPU (thermoplastic polyurethane) [14,18]. While TPEs are widely used as rubber-like materials in combination with more rigid materials, certain demanding applications exceed the TPEs’ limits for chemical resistance, thermal stability, and mechanical performance. In such cases, fully cured thermoset rubbers offer a superior alternative.

Combining thermoset rubber with thermoplastics in a single process has traditionally been challenging because of two main considerations. First, the essential processing requirements for each type of material are quite different [19]. For example, in injection molding, thermoplastics are injected at high temperatures into a cooled mold to solidify, while thermosets are injected at low temperatures into a heated mold to cure (solidify). Second, factors inherent to the materials, such as the solubility parameters, limit the thermoset–thermoplastic adhesion strength. In fact, the information available on the compatibility between thermoset rubbers and thermoplastics by overmolding can be considered limited compared to thermoplastics overmolding. Moreover, only a few studies have evaluated processing behavior and the effect of processing parameters on the adhesion between fully compounded thermoset elastomers and thermoplastics [20].

Thermoset rubber and thermoplastics have been combined by manually assembling separate components and overmolding processes; however, these parts have limitations, such as the risk of incorrect assembly or limited geometry. Bex et al. published a two-component injection molding method with thermally separated mold cavities in which high-density polyethylene (HDPE) and EPDM were combined in a single process [19]. Recent advances in MMAM have also enabled the combination of thermoplastics with thermosets, such as PLA (polylactic acid) with PDMS (polydimethylsiloxane), through hybrid systems integrating fused filament fabrication (FFF) and direct ink writing (DIW), or acrylate-based photopolymers combined with TPU through novel techniques like fusion jetting (FJ) [16,18]. Leinweber et al. reported a 3D printing system that combines a co-rotating twin screw unit and an FFF-head, enabling the printing of carbon black-filled rubber and thermoplastic [21]. The thermoplastic acted as a shell to maintain the geometry of the rubber during printing and curing, although multi-material parts were not studied in this research.

Our work investigates a dual-head extruder system to overcome the limitations of printing compounded rubbers in combination with thermoplastics. The system features a piston-based extruder for rubber and a filament-based extruder for thermoplastics, enabling the fabrication of high-quality multi-material parts in a single print. This approach allows the production of components not possible through traditional manufacturing and provides a platform to study the factors that influence interfacial adhesion between thermoplastics and thermoset rubbers. We characterize adhesion between fully compounded NBR and four thermoplastics, evaluating the effect of material-intrinsic compatibility. A representative material pair was then selected to study the influence of processing conditions, including curing and printing temperatures (via tensile testing) and raster orientation (via peel testing). Interfacial behavior was further examined through scanning electron microscopy (SEM) and energy-dispersive X-ray spectroscopy (EDS) at the fracture surface. The goal is to enable both academic and industrial applications to incorporate this novel material combination.

## 2. Materials and Methods

### 2.1. Materials

One thermoset and four thermoplastic materials were chosen for this study. Four thermoplastics, as presented in Table 1, were selected based on three criteria. First, the heat deflection temperature should exceed 130 °C, which was the rubber curing temperature post-printing. Second, the printing temperature had to remain below 300 °C because of the printer’s upper limit. Last, a diversity of amorphous and semi-crystalline materials was selected to evaluate the effect of material morphology and surface energy.

The formulation for the compounded thermoset elastomer is presented in Table 2 and is based on an optimized printable formulation created by this research group [9]. Nitrile butadiene rubber (NBR) (high and low molecular weight) was the base elastomer (high molecular weight (Nipol DN2835, ZEON Chemicals, Louisville, KY, USA) and low molecular weight (Nipol 1312, ZEON Chemicals, Louisville, KY, USA)). Carbon black (N330 Carbon Black, Tokai Carbon CB Ltd., Borger, TX, USA) was used as a reinforcing filler. A sulfur cure package was used consisting of sulfur (Rubber Maker, Harwick Standard, Akron, OH, USA) with N-cyclohexyl benzothiazole-2-sulfenamide (DURAX (CBS) Powder, Vanderbilt Chemicals LLC, Norwalk, CT, USA) as the accelerator, along with zinc oxide (ZOCO102^®^, Zochem Inc., Brampton, ON, Canada) and stearic acid (A292-500^®^, Fisher Chemical, Toronto, ON, Canada).

Two different mixing procedures were used. First, the rubber formulation used for the adhesion testing between NBR and the four different thermoplastics, along with the NBR for the peel test, were compounded in a Brabender Plasticorder Intelli-Torque Plus (Model 01-55-000, Brabender, Duisburg, Germany) internal mixer with counter-rotating screws, in batches of 50 g. The mixing process began by kneading a combination of high molecular weight and low molecular weight rubber for 2 min. Carbon black was then added, and the mixture was blended for an additional 5 min for proper dispersion. The remainder of the constitutive materials were incorporated in the order specified in Table 2. After adding sulfur, mixing continued until the torque stabilized, indicating proper mixing. The total mixing time ranged from 18 to 20 min, and the mixing temperature was kept at 65 °C.

Second, the rubber compounded for the tensile test between NBR and PC to evaluate the effect of processing conditions on adhesion was created in two-kilogram batches using a Farrel Banbury rubber mixer (Serial 64A768, Farrel Corporation, Ansonia, CT, USA). The mixing process commenced with the high molecular weight NBR, which was processed for 2 min. Subsequently, the low molecular weight NBR and the remaining components except sulfur were added, and mixing continued for an additional 4 min to ensure the carbon black was properly dispersed. Finally, sulfur was incorporated into the compound, followed by 2 more minutes of mixing. The total mixing duration was 8 min, with a mixing temperature maintained at 65 °C.

### 2.2. Curing Behavior Study

An oscillating disk curemeter (Rubber Process Analyzer 2000, Alpha Technologies, Hudson, OH, USA) was employed to examine the rubber compound’s rheological properties and curing behavior at various printing and oven curing temperatures. For each analysis, five grams of uncured rubber were placed in the RPA, and the tests were conducted in accordance with ASTM standard D2084 [22]. The cure profile was assessed at different printing temperatures (90 °C, 100 °C, and 110 °C) and oven curing temperatures (120 °C, 130 °C, and 140 °C). The available processing window before the rubber began to cure and the necessary curing time for the printed samples were determined, as well as the curing time. The frequency was maintained at 100 CPM, and the rotational amplitude was set to 1° for all measurements.

### 2.3. Multi-Material 3D Printing

**Figure 1:**
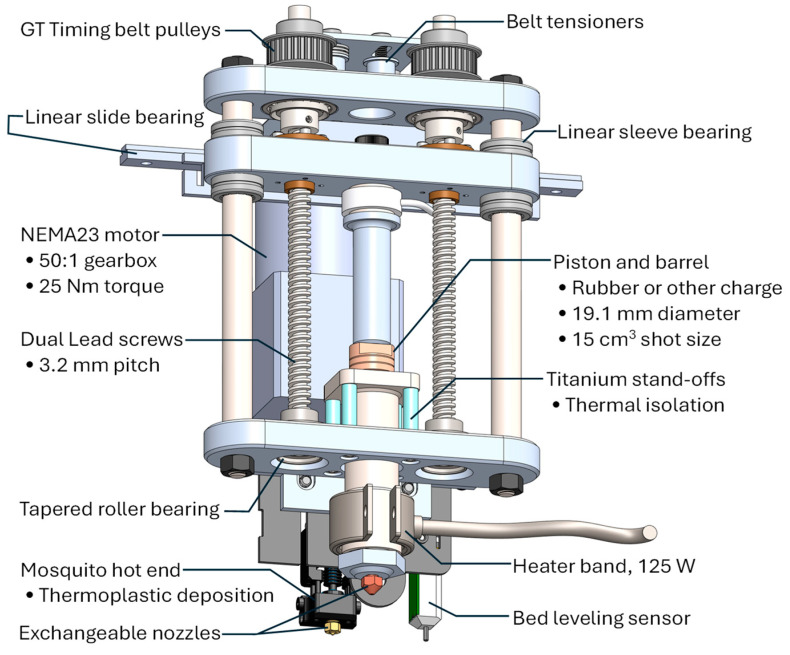
Layout design of the enhanced Additive Ram Material Extruder (ARME) for multi-material printing with traditional thermoplastic filament and fully compounded rubber.

Multi-material printing was performed using an Ender 5 Pro (Creality, Shenzhen, China) modified with a custom print head, ARME 3XL, an enhanced version of the Additive Ram Material Extruder (ARME), which was originally developed for 3D printing with extrusion of fully compounded nitrile rubber [8]. Depicted in Figure 1, the ARME 3XL integrates a NEMA23 motor with a 50:1 gearbox that drives dual leadscrews (3.2 mm pitch) at speeds up to 18 RPM. A 19.1 mm diameter piston drives the extrusion through a barrel with a 62 mm shot length, delivering a maximum shot volume of 15 cm^3^. Each motor step (10,000 steps/rev) corresponds to 3.2 nm displacement and 0.0916 mm^3^ output, allowing precise material control. At 25 Nm torque, the extruder generates 18.6 kN of compressive force corresponding to a maximum extrusion pressure of 650 bar. Given this transmission design is directed to high force at low displacement, however, the maximum ram speed is 1.92 mm/s with a maximum flow rate of 0.55 cm^3^/s.

The extrusion barrel is heated with a 125 W band heater with PID control via feedback from a type K thermocouple. A Mosquito hot end (Slice Engineering, Gainesville, FL, USA) with a bimetallic heat break, comprising a copper alloy and strain-hardened steel, enables extrusion of materials up to 500 °C. Both the barrel and Mosquito hot end can fit with a variety of exchangeable nozzles having diameters ranging from 0.2 to 3 mm. The entire assembly weighs 3.6 kg and is top-mounted on the Ender 5 Pro’s central rail with its center of mass positioned below the linear slide bearing to ensure mechanical stability. All motion and extrusion functions were fully operable with the stock Creality controller and firmware, with custom g-code enabling synchronized extrusion volumes based on calibrated motor steps.

Both thermoplastic and thermosetting resins have been processed at a variety of temperatures. The printing process begins with loading the rubber in the barrel piston, heating it under light compression for 5 min, and then starting the printing process of the desired dual material shape. The printing conditions are reported in Table 3.

**Figure 2:**
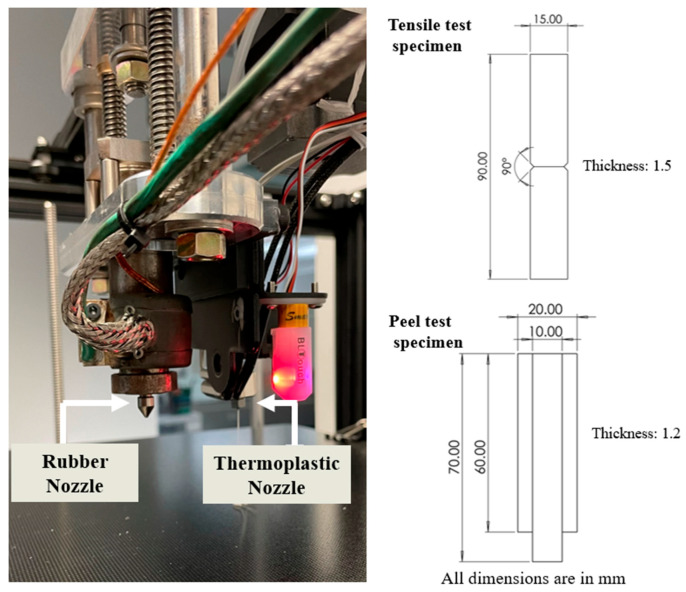
ARME 3XL print head and engineering drawing for tensile test and peel test specimens.

Two geometry types were printed for the adhesion tests. The geometries and dimensions are illustrated in Figure 2, as well as the ARME 3XL print head. For the tensile test, two materials were printed into a rectangular bar with a joint in the middle. A notch (angle of 45 degrees) was created at the joint of the two materials to ensure failure at the bonding area between the thermoset and thermoplastic for measuring the adhesion strength between the two materials. The printing pattern was perpendicular to the tensile direction. For the peel test, specimens were printed according to ASTM D429 [23] with a scale modification. The samples were printed using two patterns: one with the raster oriented perpendicular to the pull direction, and the other with the raster oriented parallel to the pull direction. Peel samples were fabricated by placing a Teflon film between the PC and rubber layers partway from the end to enable separation of the layers to create a peel sample. After printing, all the specimens were oven-cured at the selected temperatures for the specified duration to achieve 90% of the cure based on the RPA data.

### 2.4. Adhesion Tests

Two types of adhesion tests were conducted to evaluate the adhesion between the thermoset and thermoplastic material sets: (1) tensile test and (2) peel test. The adhesion strengths between the thermoset and the thermoplastic materials listed in Table 1 were evaluated through tensile testing using a universal testing machine (Instron 4466, Norwood, MA, USA) at a crosshead speed of 50 mm/min. A 50 N load cell was used. Three samples of each combination of NBR with a thermoplastic were tested. The adhesion strength, defined as the maximum stress, was calculated by dividing the maximum force by the measured joint area.

After evaluating all the thermoplastics in combination with the NBR, polycarbonate was selected to understand the effects of the printing and curing process parameters on the adhesion strength between the thermoset and the thermoplastic, specifically the printing temperature and the oven curing temperature for the rubber. The process conditions are presented in Table 4.

The peel testing was conducted following a modified version of ASTM D429 to assess the adhesion of rubber to rigid substrates. Method B was selected, which specifies a 90° peel test that measures the force required to peel rubber from a rigid substrate (usually metal) at a 90-degree angle, specifically polycarbonate in this case. The dimensions were modified to optimize the printing time of the specimens (Figure 2). The specimens were evaluated using a single-column Instron 68SC-05 equipped with a 90-degree test fixture and a 500N load cell. The thermoplastic was secured in the fixture while the rubber was clamped. The separation of the rubber was set at a 50 mm/min rate until it was completed. The adhesion value was obtained by calculating the average force required to peel the rubber from the thermoplastics over a defined displacement range, divided by the width of the rubber strip.

### 2.5. SEM and Energy-Dispersive X-Ray Spectroscopy

Scanning electron microscope (SEM) images of the failure interface in the NBR–PC printed samples, taken after tensile testing, were obtained using a Field-Emission Scanning Electron Microscope (FESEM) (JSM 7401F, JEOL Inc., Peabody, MA, USA). Energy-dispersive spectroscopy (EDS) analysis was conducted to map and identify the elemental composition of the interface of the samples after the tensile test (point of failure). Knowing the elemental composition of the materials allows for assessing the presence of elements from the materials present on the failure surface.

## 3. Results and Discussion

The printing and adhesion of the samples are influenced by various factors, including the material, its viscosity, and the curing process, among others. The rheological properties of the elastomer at different temperatures will vary due to its curing behavior. A detailed description of the cure rheology is first described, followed by the structural characterization results.

### 3.1. Rheology and Cure of NBR Compound at Different Printing and Curing Temperatures

To determine the processing parameters of thermoset rubber, it is essential to observe the crosslinking and rheological behavior of the material. The curing process involves the chemical formation of crosslinks (in this system, sulfur) between the long polymer chains, resulting in a thermally induced three-dimensional elastic network [24,25,26]. In industry, the oscillating disk rheometer is frequently used to characterize the curing process of rubber, as specified in ASTM D2084-19a [22]. During this process, the necessary torque required for a sample to achieve a certain amplitude at a fixed temperature is measured, directly associating the torque with the units of crosslinks formed per unit volume of rubber [24]. The torque plotted against time is the cure curve.

The Rubber Process Analyzer (RPA) was utilized to obtain the cure curves for each processing temperature, determining the time before crosslinking initiates and the duration of the crosslinking process. These factors were crucial for setting up the additive manufacturing of the compounded NBR. First, the initial formation of the three-dimensional network was considered. From this point forward, the compound ceases to flow and cannot be shaped, which corresponds to the Mooney Scorch time (ts2) defined as the required time for the cure state to increase by two torque units above the minimum torque (ML) [26]. Experimentally, ts2 defines the processing window available for the NBR at the selected temperatures, and, therefore, the time available for printing after the barrel of the ARME printer was heated.

After printing the samples, the curing process was carried out in an oven. The optimum cure time (t90) is the duration needed to achieve 90% of the difference between the minimum torque (ML) and the maximum torque (MH) [26,27]. This time is typically chosen for curing the samples as it results in optimal physical properties by preventing overcure and reversion of the compound. Therefore, to study the effect of three different curing temperatures on the adhesion strength between a thermoset and a thermoplastic, the t90 for each was used as the curing time, ensuring an optimal level of curing and properties for each sample.

**Figure 3:**
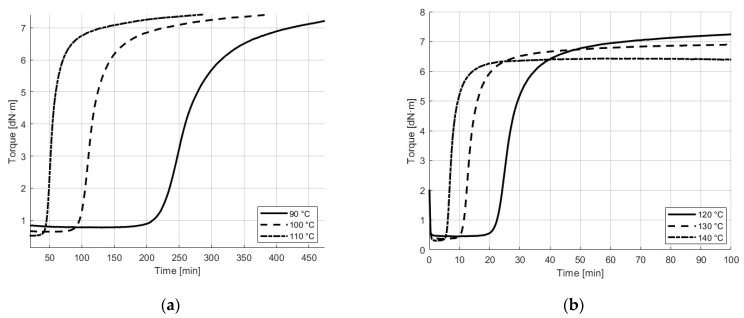
Cure behavior of NBR compound (**a**) at printing temperatures and (**b**) at oven curing temperatures.

The first adhesion section of this research studied the adhesion of different thermoplastics with NBR printed at 100 °C and cured at 130 °C. This was followed by a second section, which studied the effects of varying the printing temperature (from 90 °C to 110 °C) and the curing temperature (from 120 °C to 140 °C) for one pair of materials. As shown in Figure 3, the cure kinetics of the NBR compound are temperature-dependent, with lower temperatures resulting in slower crosslinking and extended cure windows. The curing parameters, such as the minimum torque (ML), maximum torque (MH), scorch time (ts2), and cure time (t90), are reported in Table 5. It is important to note that the viscosity of rubber is often associated with the torque produced by an oscillating cure meter, and the values can be discussed interchangeably [9]. Therefore, in this work, viscosities are evaluated in torque units.

As expected, increasing the temperature reduced the printing window (ts2), as well as the curing times (t90), for the NBR formulation. When evaluating the minimum torque (ML) at the printing temperatures, it is noted that the highest viscosity (0.8 dN·m) occurs at the lowest temperature (90 °C), and it decreases as the temperature rises. The same trend is observed for the maximum torque (MH), indicating that at higher curing temperatures, the viscosity will be lower compared to lower curing temperatures. This behavior is used to understand the effect of viscosity and cure on the adhesion with the thermoplastic.

### 3.2. Tensile Adhesion Test Results

To understand the effect of the material on adhesion strength, NBR combined with the four thermoplastics was investigated. Then, one material set was picked to study the effect of the processing conditions.

#### 3.2.1. NBR with Thermoplastics

Four thermoplastics were selected to be printed with NBR, evaluating the adhesion strengths between the materials and the inherent factors affecting the bond strength. The adhesion strengths of the four pairs of materials were characterized by dividing the peak force by the actual cross-sectional area of each sample, thus normalizing the adhesion strength. The results of the adhesion strengths between the NBR compounded rubber and the thermoplastics are presented in Table 6. The reported uncertainties correspond to the standard deviation obtained from three independent replicate tests for each sample configuration.

The adhesion strength between NBR and PC was the strongest among the selected materials, followed by PVDF, PA, and HTPLA. Polymer-to-polymer adhesion is a complicated process affected by multiple parameters [28]. Here, we chose to examine the parameters we felt were most relevant: the solubility parameter, mobility (viscosity), and morphology (crystalline or amorphous). Both thermodynamics (free energy of mixing) and kinetics (mobility) play a role in the adhesion behavior of two polymers.

First, we examined the effect of the solubility parameter of the individual materials on the adhesion strength. For polymers to be miscible, the free energy of mixing (∆G) must be negative. However, ΔG, which is equal to the change in enthalpy (ΔH) minus the temperature times the change in entropy (ΔS), tends to be positive for polymers due to the contribution of these terms. The enthalpy is normally greater than zero, while the entropy of mixing is typically very small for high molecular weight materials, making polymers unlikely to intermingle [29,30,31]. Despite this, to minimize interfacial energy, statistical thermodynamic theories predict that different degrees of interdiffusion will occur at the interfacial layer [32,33,34]. The closer the Hildebrand solubility parameters are, the more likely the materials will have affinity for each other, resulting in greater interdiffusion at their interface [35].

Among many models and theories, the Hansen or partial solubility parameter (HSP), an enhanced predictive parameter derived from the Hildebrand total solubility parameter, considers the effect of the three major modes of interaction that contribute to the cohesive energy between two polymers: dispersion forces (δD), polar forces (δP), and hydrogen bonding (δH) [36,37]. The location of solvents can be plotted on a δP versus δH graph, and the distances between points (Ra) can be calculated using Equation (1) [38,39]. In addition to the Hansen distance, the Relative Energy Distance (RED), which is a useful parameter in assessing miscibility, was calculated using Equation (2). For this, the Hansen distance (Ra) is divided by the Hansen solubility parameter sphere (Ro) [38,39]. The calculated Ra and RED values are presented in Table 7.

(1) $${Ra}^{2}=4{\left(\delta D1-\delta D2\right)}^{2}+{\left(\delta P1-\delta P2\right)}^{2}+{\left(\delta H1-\delta H2\right)}^{2},$$

(2) $$RED=\frac{Ra}{Ro},$$

The dispersion, polar, and hydrogen bonding parameters are also specified in Table 7. Since the exact solubility parameters of the commercial materials are unknown, literature references were used as a reasonable approximation for the selected materials. For PC, PA, and PVDF, solubility parameters were included from the Hansen Solubility Parameters User’s Handbook [35], a widely accepted reference that compiles experimentally derived values from multiple sources. NBR and HTPLA are not reported in the Hansen compilation. Therefore, solubility parameters for both NBR and HTPLA were extracted from two independent literature sources to ensure reliability. Particularly for the HTPLA, which is a commercially modified version of Poly(Lactic Acid) (PLA), standard PLA solubility parameters were utilized.

According to theory, materials with a Ra close to zero and a RED value below one are considered thermodynamically miscible [42]. This is consistent with the results for NBR and PC, which showed the strongest adhesion, along with the lowest Ra (2.29–2.70 MPa^1/2^) and a RED below one (0.19–0.22). PVDF and PA also followed the trend. Their higher Ra and RED values above one indicated lower miscibility with NBR, which matched their lower adhesion strength. The only exception was HTPLA. Although it had the second lowest Ra and a RED value near zero, both indicating miscibility, it exhibited the weakest adhesion with NBR. This discrepancy likely arises from the proprietary formulation of HTPLA, which may contain additives or structural modifications not considered in standard PLA solubility parameters. Since the exact Hansen solubility parameter of the commercial HTPLA used in this study is unknown, the predicted miscibility based on PLA literature values might not accurately reflect the actual interfacial behavior. While experimental determination of the Hansen solubility parameters through solvent interaction tests would provide more precise insight, this was not conducted in this research.

**Figure 4:**
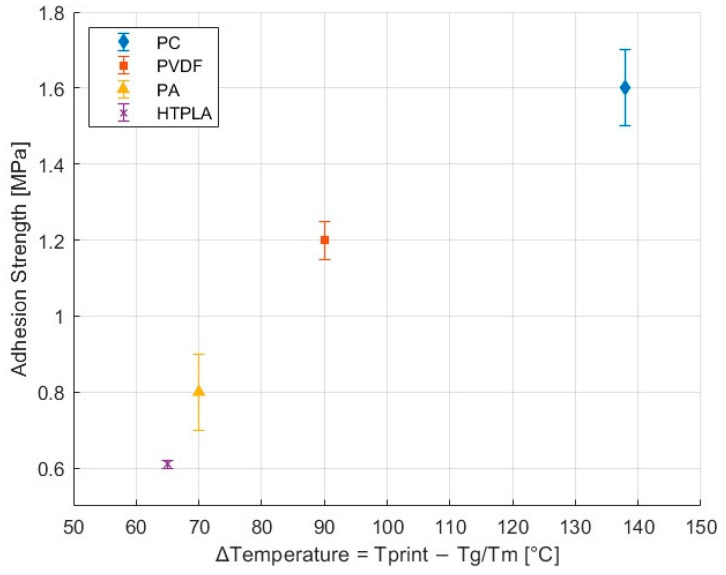
Adhesion strength between NBR and selected thermoplastics as a function of the temperature difference (ΔT) between the printing temperature and the highest transition temperature of each material. For amorphous polymers, ΔT = Tprint − Tg; for semicrystalline polymers, ΔT = Tprint − Tm.

Another key factor is chain mobility or viscosity. In amorphous materials, chain mobility occurs when the temperature exceeds the glass transition temperature (Tg), allowing the polymer chains to acquire sufficient energy to move and diffuse, as is the case with PC. In contrast, semicrystalline polymers contain crystalline domains that limit chain mobility below the melting temperature; thus, it is crucial for the temperature to rise above the melting point for mobility to occur [44]. Given potential miscibility, mobility is also required for interdiffusion to occur across the interface and provide good adhesion. Therefore, the difference between the printing temperature and Tg and Tm for the amorphous and semicrystalline materials, respectively, was determined. Greater differences between the printing temperature and the highest polymer’s transition temperature (either Tg for amorphous or Tm for semicrystalline materials) resulted in greater adhesion strength, a trend clearly observed in Figure 4, which is likely due to enhanced chain mobility and increased interfacial bonding time. Notably, the higher adhesion strength of NBR with PC corresponded to the largest delta between the printing temperature of PC and its transition temperature. The second highest adhesion strength was noted for PVDF, which also had the second-largest delta between these temperatures. This relationship was similarly reflected in PA and HTPLA, which both exhibited the lowest adhesion strength with NBR among the evaluated material pairs.

PC was the only amorphous polymer among the tested thermoplastics and was also the one that provided the highest adhesion. The adhesion mechanism is complicated and varies between amorphous and semicrystalline polymers. In the case of amorphous polymers, interdiffusion is more straightforward as it depends on diffusion and entanglements, both of which happen freely in a molten state. However, for semicrystalline polymers, the mechanism is more complex, and the crystallization plays a crucial role that can hinder interdiffusion, since only a single material can exist in the crystalline portion [45].

The measured interfacial adhesion strengths between NBR and the thermoplastics ranged from 0.6 MPa (HTPLA) to 1.6 MPa (PC). The tensile properties of the same NBR formulation were previously evaluated [9]. In particular, specimens printed with roads transverse to the loading direction, the most relevant for interfacial failure, showed an ultimate tensile strength of 8.4 ± 0.5 MPa. Therefore, the highest measured adhesion strength (1.6 MPa for NBR–PC) corresponds to ~19% of the transverse strength of the rubber. While modest, these values could be improved with modified process conditions, designs, or material modifications (e.g., adhesion promoters).

#### 3.2.2. Effect of Processing Parameters on the Adhesion Strength of NBR and PC

In the previous section, we studied the effect of factors inherent to different material sets on adhesion strength. In this section, the best pair of materials, NBR and PC, was selected to study the effect of the processing parameters on this bond. In this work, only the processing parameters for the rubber were varied, while the thermoplastic (PC) processing parameters were kept constant. Two main conditions were varied for the NBR: the printing temperature (PT) and the rubber curing temperature (CT). Table 8 shows the values used for both the printing and curing temperatures, as well as the adhesion strength.

Before discussing the results, it is relevant to note that this research explores the adhesion between fully compounded thermoset rubber and thermoplastics in an additive manufacturing context, which does not appear to be previously studied. Therefore, the existing literature on rubber–thermoplastic overmolding and thermoplastic adhesion was used to establish a comparative framework for interpreting the results.

**Figure 5:**
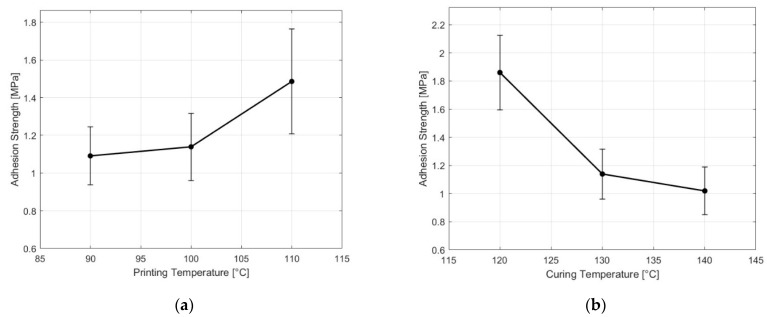
Effect of the (**a**) printing temperature and (**b**) curing temperature on the adhesion strength between NBR and PC.

First, the adhesion strength between fully compounded NBR and PC was evaluated as a function of the rubber printing temperature, ranging from 90 °C to 110 °C. Figure 5 shows a modest increase in average adhesion strength when the printing temperature increases. This limited effect contrasts with studies on thermoplastics, where increasing nozzle or melt temperature has been shown to enhance interfacial adhesion by reducing viscosity and promoting chain interdiffusion [20,44,46,47,48]. For instance, Yin et al. [44] reported a 15.3% increase in adhesion strength between TPU and ABS as the nozzle temperature increased from 240 °C to 260 °C.

Although increasing the temperature reduces the viscosity of NBR (Table 5), the printing temperature range studied (90 °C to 110 °C) is low compared to the glass transition temperature of PC (147 °C), making it unlikely to melt the PC during the printing process. The nozzle’s brief contact time further limited interfacial heating. This is consistent with findings by Bex et al. [19], who observed a minimal impact of injection temperature on adhesion in overmolding sulfur-based rubber onto thermoplastics, as the low injection temperature for rubber did not sufficiently increase the interface temperature to enhance bonding [49]. Additionally, variability in the measured values increased as the printing temperature rose. One reason for this is that the printing window, before curing, becomes narrower at the highest temperatures, resulting in very little time to complete the print (which is impractical for actual processes).

The second parameter studied was the rubber curing temperature, which influenced adhesion more significantly than the printing temperature. Increasing the curing temperature from 120 °C to 140 °C led to a 47% decrease in average adhesion strength. This outcome contrasts with overmolding studies, where higher temperatures generally enhance adhesion [49,50]. However, in our study, curing times were determined based on t90, which is the typical point at which most rubber is considered to have optimal physical properties (to avoid overcure due to the insulative nature of the rubber). As a result, the curing times varied significantly for each temperature: 50 min for 120 °C, 19 min for 130 °C, and 13 min for 140 °C. Although rubber viscosity is higher at 120 °C, the longer curing duration allows more time for chain interdiffusion, leading to stronger adhesion.

This behavior aligns with reptation theory by de Gennes [20], which predicts increased interfacial strength with longer diffusion times, and with Aradian, Raphaël, and de Gennes [51], who described how advancing crosslinking reduces chain mobility, creating a competition between network formation and interdiffusion that ultimately halts further penetration once curing restricts motion. In other words, a longer window before significant crosslinking provides more opportunity for polymer chains to diffuse across the interface and form entanglements.

Prager and Tirrell [52] further characterized the non-linear nature of this time dependence. Initially, short chains and chain ends dominate diffusion, resulting in a t^1/4^ scaling of interfacial strength. As diffusion time increases, longer chains progressively contribute to the interfacial entanglement, transitioning the strength growth toward a t^1/2^ dependence. Together, these theories support our finding that extended curing times favor better adhesion, even at moderate temperatures.

### 3.3. Raster Orientation Effect on the Adhesion Strength Between NBR and PC

The effect of printing raster orientation on the adhesion between NBR and PC was also investigated. The peel test specimens involved printing two layers of NBR on top of two layers of PC, with all layers having the same raster orientation. Two configurations were evaluated: one where the raster lines were parallel to the pull direction (0°), and another where they were perpendicular (90°). Table 9 reports the adhesion strength for both raster orientations, which is the average force over the peel length per millimeter of rubber width.

Although specimens with raster lines oriented perpendicular to the peel direction (90°) exhibited a higher average peel strength (1.2 N/mm) compared to those printed with rasters parallel to the pull direction (0.8 N/mm), the difference falls within the margin of experimental error. The overlap in standard deviations (±0.2 for 90°, ±0.3 for 0°) suggests that this increase may not be statistically significant under the tested conditions. However, the observed trend is consistent with prior studies that reported that 3D printed specimens with 90° raster orientation (perpendicular to peel direction) were more difficult to peel compared to 0° and ±45° orientations [53].

**Figure 6:**
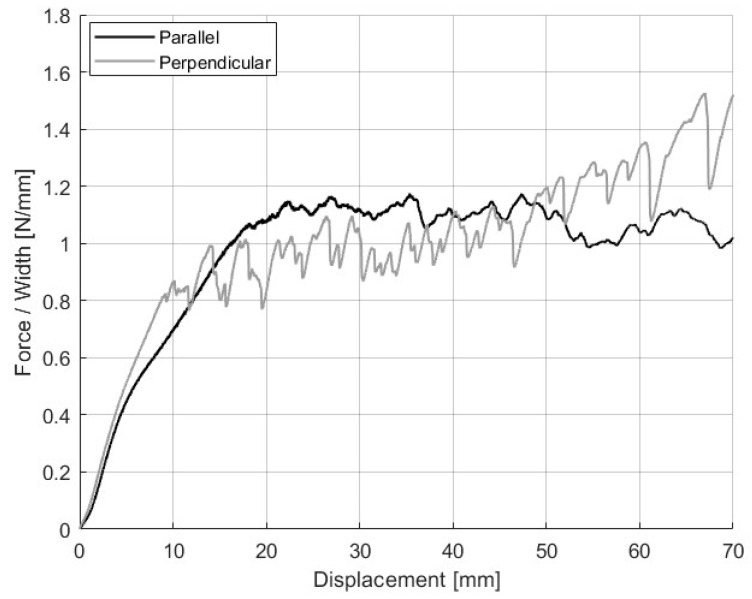
Peel tests between NBR and PC with different raster orientations.

The anisotropy introduced by the filament deposition direction during printing is shown as a higher average peel force and greater variability in the 90° samples. Their force–displacement profiles were notably less steady than the 0° orientation, as shown in Figure 6. These fluctuations are likely caused by local variations in interfacial contact between the NBR and PC layers. In the 90° configuration, the NBR raster lines intersect the PC surface at discrete intervals, resulting in a non-uniform contact area along the peel path. Specifically, some regions achieve complete contact at the interface, while others contain inter-raster voids or partial bonding due to gaps between layers. As the effective contact area varies along the specimen, the peel resistance also fluctuates, reflecting alternating zones of stronger and weaker adhesion.

While specific data on NBR–PC adhesion using conventional methods is scarce, related studies provide useful benchmarks. For instance, surface treatment of PC through ultraviolet C irradiation has been demonstrated to enhance bonding with liquid silicone rubber (LSR) in overmolding processes, resulting in peel strengths of up to 5.8 N/mm in PC-LSR systems [54]. For further context, adhesion-modified thermoplastic elastomers (TPEs), which are commonly used in the automotive and engineering sectors for overmolding soft materials onto rigid substrates, reported peel strengths of up to 5.5 N/mm when bonded to PC [55]. These values are considered excellent for soft thermoplastics. In comparison, our system achieved a maximum average adhesion peel strength of 1.2 N/mm without the use of treatments, mechanical interlocking, or adhesives. Considering the fully additive nature of the process and the challenging material pairing, this result is promising. Moreover, our approach enables the direct integration of fully compounded rubber with thermoplastics, offering design freedom unattainable by conventional methods. Based on our findings, the resulting interfaces may be suitable for applications such as gaskets, seals, or soft–rigid components requiring moderate adhesion. Future work may involve surface activation or mechanical features to enhance bonding further.

### 3.4. SEM and Energy-Dispersive X-Ray Spectroscopy (EDS)

**Figure 7:**
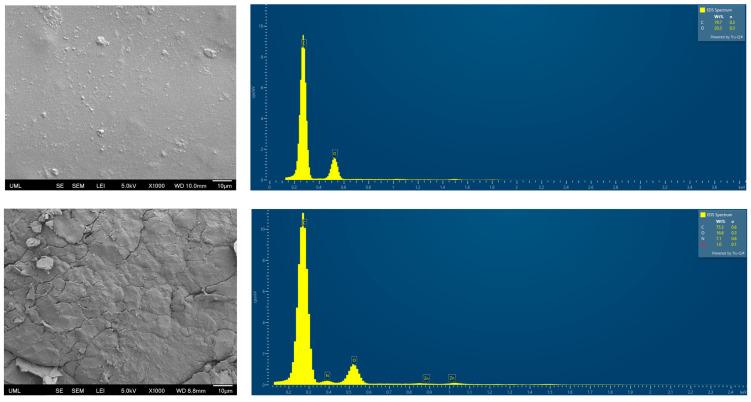
SEM (**left**) and EDS (**right**) images taken on the PC side of the fractured sample after tensile testing. (**top**) Region far from the NBR–PC interface; (**bottom**) region near the NBR interface.

SEM imaging was performed at the failure interface after tensile testing, focusing specifically on the PC side of the fractured samples. The objective was to assess whether any elements characteristic of the NBR compound were present on the PC surface, which would suggest potential interdiffusion across the interface. Two SEM-EDS images are shown in Figure 7, both taken from the PC surface: the first from a region distant from the cross-section, and the second from the edge adjacent to the NBR layer. In the distant region, only carbon and oxygen were detected, consistent with the expected elemental composition of polycarbonate. In contrast, the image taken near the NBR interface revealed the presence of nitrogen and zinc, elements not native to PC, but associated with the NBR formulation. Nitrogen is present in the chemical structure of NBR as well as in N-cyclohexylbenzothiazole sulfenamide (CBS), which was used as the accelerator in the formulation. Zinc was introduced by adding zinc oxide (ZnO), employed as an activator in the sulfur-based curing system. The presence of these elements on the PC side suggests a degree of interfacial interdiffusion between the NBR and PC, enhancing the bonding between the two materials. While this analysis is qualitative and does not provide information on the diffusion depth, it nonetheless offers useful insight into the presence of NBR-related elements at the PC surface. To further quantify interfacial diffusion and establish a more direct correlation with adhesion strength, additional characterization techniques, such as X-ray Photoelectron Spectroscopy (XPS), could yield valuable insights in future studies.

## 4. Conclusions

This study addressed the challenge of integrating fully compounded thermoset elastomers with high-performance thermoplastics via multi-material 3D printing. The objective was to investigate interfacial adhesion in printed thermoset–thermoplastic composites by evaluating both the material-intrinsic compatibilities across four thermoplastics and the influence of key processing parameters on a selected pair.

A dual-extrusion system was successfully employed to fabricate bonded structures of NBR rubber and four different thermoplastics. Among the materials tested, polycarbonate (PC) exhibited the highest adhesion strength with NBR, correlating with favorable solubility parameter proximity, amorphous morphology, and enhanced chain mobility at the processing temperature.

Curing conditions significantly influenced adhesion strength. Lower temperatures for longer durations (e.g., 120 °C for 50 min) outperformed short, high-temperature ones (e.g., 140 °C for 13 min), likely due to increased interdiffusion before full crosslinking. Future research will systematically explore how other key printing parameters, such as speed, layer height, and extrusion pressure, affect interfacial adhesion. These variables are likely to have a significant impact on the interactions at the interface during dual-material printing, and focusing on them could improve adhesion quality and manufacturing reliability.

Adhesion strength was also influenced by raster orientation, with perpendicular alignment (90°) showing stronger adhesion than parallel raster alignment (0°). Lastly, SEM-EDS confirmed the migration of rubber-specific elements to the thermoplastic surface, indicating interfacial interaction.

Together, these findings provide a framework for understanding and optimizing the adhesion between fully compounded rubbers and thermoplastics in additive manufacturing, expanding the design space for next-generation multi-functional parts.

## Figures and Tables

**Table 1:** Thermoplastics.

Material	Brand Name	Heat Deflection Temperature (°C)	Printing Temperature (°C)	Bed Temperature (°C)
Polycarbonate (PC)	3DXMax PC (3DXTECH, Grand Rapids, MI, USA)	135	275–295	90
Polyvinylidene Fluoride (PVDF)	FluorX™ PVDF (3DXTECH, Grand Rapids, MI, USA)	158	255–275	80
High-Temperature Polylactic Acid (HTPLA)	Proto-pasta HTPLA (Protoplant, Vancouver, WA, USA)	140	200–210	60
Polyamide 6 (PA6)	AmideX PA6 Copolymer (3DXTECH, Grand Rapids, MI, USA)	140	270	80

**Table 2:** Formulation For NBR compounds.

Component	Parts per Hundred Rubber (PHR)
High molecular weight NBR	50
Low molecular weight NBR	50
Carbon Black N330	60
Zinc oxide	4
CBS	2
Stearic acid	0.5
Sulfur	1

**Table 3:** Printing parameters.

Component	Thermoset	Thermoplastic
Printing speed (mm/s)	10	12
Nozzle size (mm)	0.8	0.4
Layer height (mm)	0.3	0.3

**Table 4:** Process parameter variations.

Variable	Printing Temperature (°C)	Curing Temperature (°C)
Control sample	100	130
Effect of printing temperature	110	130
	90	130
Effect of curing temperature	100	140
	100	120

**Table 5:** Cure parameters for NBR compound at print and cure temperatures.

Material	90 °C	100 °C	110 °C	120 °C	130 °C	140 °C
Minimum torque, ML (dN·m)	0.8	0.6	0.5	0.5	0.4	0.3
Maximum torque, MH (dN·m)	7.3	7.6	7.6	7.5	6.9	6.4
Scorch time, ts2 (min)	245	108	51	25	13	7
Cure time, t90 (min)	359	200	109	50	19	13

**Table 6:** Adhesion strengths between NBR and selected thermoplastics.

NBR with	Adhesion Strength (MPa)
PC	1.6 ± 0.1
PVDF	1.2 ± 0.1
PA	0.8 ± 0.1
HTPLA	0.6 ± 0.0

**Table 7:** Solubility parameters of selected materials, and thermoplastics listed by Ra and RED number relative to NBR.

Polymer	δD (MPa^1/2^)	δP (MPa^1/2^)	δH (MPa^1/2^)	Ro (MPa^1/2^)	Ra (MPa^1/2^)	RED	Adhesion Strength (MPa)
NBR ^1^	19.6	8.7–9.6	6.3–6.7	-	-	-	
PC ^2^	19.1	10.9	5.1	12.1	2.29–2.70	0.19–0.22	1.6 ± 0.1
PVDF ^2^	17	12.1	10.2	4.1	6.75–7.34	1.65–1.79	1.2 ± 0.1
PA ^2^	17	3.4	10.6	5.1	8.58–8.98	1.68–1.76	0.8 ± 0.1
HTPLA ^3^	17.9–18.6	9–9.9	5.9–6	10.7	2.14–3.54	0.20–0.33	0.6 ± 0.0

^1^ See Refs. [40,41]. ^2^ See Ref. [35]. ^3^ See Refs. [42,43].

**Table 8:** Effect of processing parameters on adhesion strength between NBR and PC.

Sample	Printing Temperature (°C)	Curing Temperature (°C)	Adhesion Strength (MPa)
Control	100	130	1.1 ± 0.2
Higher PT	110	130	1.5 ± 0.3
Lower PT	90	130	1.1 ± 0.2
Higher CT	100	140	1.0 ± 0.2
Lower CT	100	120	1.9 ± 0.3

**Table 9:** Peel peak strength between NBR and PC.

Print Direction (°)	Description	Average Force/Width (N/mm)
0	Raster lines parallel to the pull direction	0.8 ± 0.3
90	Raster lines perpendicular to the pull direction	1.2 ± 0.2

## Data Availability

The data presented in this study are contained within the article. Further inquiries can be directed to the authors.
